# Effect of strategic nutrient reduction and exogenous enzyme supplementation on mineral and energy balance in growing pigs

**DOI:** 10.5713/ab.25.0568

**Published:** 2025-09-30

**Authors:** Gerardo Ordaz, Sergio Gómez, María de Lourdes Angeles, Maria Alejandra Pérez

**Affiliations:** 1 Centro Nacional de Investigación Disciplinaria en Fisiología y Mejoramiento Animal, Instituto Nacional de Investigaciones Forestales Agrícolas y Pecuarias, Ajuchitlán Colón, QT, México

**Keywords:** Energy Balance, Exogenous Enzymes, Minerals, Precision Nutrition, Sustainability

## Abstract

**Objective:**

This study evaluated the effects of strategic nutrient reduction in combination with exogenous enzymes (phytase, pectinase, and β-glucanase) on mineral and energy balance in growing pigs.

**Methods:**

Twenty-four barrows (age: 97±5 days) with an average initial body weight of 49.26±0.42 kg were individually housed in metabolism crates under controlled conditions. Diets were offered for 10 days, consisting of a 5-day adaptation period followed by a 5-day total-collection balance period. Pigs were assigned to four experimental treatments: a positive control diet (PC-100) with standard levels of metabolizable energy (ME), calcium, phosphorus, and mineral premix; a negative control diet (NC-100) with the same mineral premix but reduced by 100 kcal/kg ME, 0.05% calcium, and 0.10% phosphorus, supplemented with the enzyme blend; and two additional diets based on NC-100 with a 33% (NC-67) and 66% (NC-34) reduction in the mineral premix. Productive performance, apparent fecal digestibility (AFD), nutrient retention, and energy efficiency were measured.

**Results:**

Results showed that mineral reduction (33% and 66% vs. the recommended level) together with exogenous enzymes did not affect daily weight gain or feed efficiency (p>0.05). However, a significant decreased total nitrogen excretion (from 13.66 to 10.72 g/day; p<0.001), and increased proportion of retained nitrogen relative to absorbed nitrogen (from 73.46% in PC-100 to 81.69% in NC-34; p = 0.0025) were observed. AFD of phosphorus improved with enzyme supplementation (up to 74.89% in NC-100; p<0.01), and zinc digestibility increased significantly with mineral premix reduction (up to 50.01% in NC-34; p<0.01). ME remained stable among treatments (p = 0.06), with average values ranging from 3,593 to 3,642 kcal/kg.

**Conclusion:**

Strategic reduction of dietary minerals (33% and 66% vs. the recommended level), and energy, in combination with exogenous enzymes, improved nutrient utilization without negatively affecting short-term growth performance in growing pigs.

## INTRODUCTION

The intensification of pig production has been a direct response to the growing global demand for animal protein; however, it has also brought significant environmental consequences, particularly associated with the formulation of diets containing micro- and macromineral levels above recommended values [1,2]. These minerals, commonly supplemented in inorganic form, are often added in excess to ensure a minimum availability that supports animal growth and health, given their limited bioavailability due to antinutritional factors present in feed ingredients [3]. This traditional formulation model, based on wide safety margins to account for physiological and technological variation, has proven inefficient from an ecological perspective. Several studies have shown that a substantial proportion of supplemented minerals is not absorbed by the animal, especially in diets based on plant-derived ingredients rich in phytates, which limit the actual availability of phosphorus and other essential divalent cations such as calcium, zinc, and iron [4,5]. This not only represents an economic loss but also increases the risk of eutrophication and heavy metal toxicity in agricultural environments [6,7], and contributes to antimicrobial resistance, as is the case for copper and zinc [8,9].

In response to this issue, nutritional strategies have been proposed based on the strategic reduction of trace minerals, combined with the use of exogenous enzymes such as phytases, pectinases, glucanases, and xylanases. These enzymes enhance the availability of nutrients trapped within the cell walls and phytates of plant-based ingredients [10–12]. Exogenous enzymes not only improve phosphorus digestibility, but also that of associated minerals, energy, and protein, enabling more efficient and cost-effective diet reformulation [13]. Moreover, the combined use of multiple enzymes may have synergistic effects on nutrient digestion and absorption [14,15].

The implementation of these strategies aligns with the principles of sustainability and circular economy promoted by international organizations, which advocate for reducing the use of critical inputs, minimizing waste, and improving the overall efficiency of livestock systems [16,17]. In this context, several studies have reported that phytase inclusion in low-phosphorus diets can maintain productive performance in pigs while reducing environmental excretion [3,18]. However, studies that combine trace mineral reduction with multi-enzyme blends under controlled metabolic conditions remain scarce, and even fewer have focused on assessing their simultaneous effects on both energy and mineral balance in pigs.

Despite growing evidence supporting the efficacy of exogenous enzymes, there remains a critical need for studies that integrate quantitative metabolic approaches—such as apparent fecal digestibility (AFD), mineral retention, and energy efficiency—under rigorous experimental conditions. Such studies would help establish safe thresholds for mineral reduction without compromising the animal’s nutritional status and validate sustainable formulations for large-scale application. Therefore, the aim of the present study was to evaluate the effects of strategic nutrient reduction in combination with a multi-enzyme complex, on energy balance and the absorption of macro- and microminerals in growing pigs.

## MATERIALS AND METHODS

The study was conducted at the Metabolic Unit of the National Research Center for Physiology and Animal Improvement (CENID-Fisiología y Mejoramiento Animal), part of INIFAP (Mexico). The experimental protocol was reviewed and approved by the Scientific and Technical Committee of the CENID-Fisiología y Mejoramiento Animal. Animal handling followed the guidelines established by the Mexican Official Standard for the production, care, and use of laboratory animals [19], as well as the *International Guiding Principles for Biomedical Research Involving Animals* [20].

### Animals, diets, and housing

A total of twenty-four barrows (Landrace×Large White; 97±5 days old; 49.26±0.42 kg initial body weight) were individually housed in stainless-steel metabolism cages (150 length×65 width×90 height cm; floor area 0.97 m^2^). Each cage was equipped with an automatic drinker, an individual feeder, a slatted floor, and separate trays for quantitative feces and urine collection. The experimental room was maintained at 22± 2.0°C. Four diets were formulated, considering the NRC [21] requirements, and the experimental treatments are described in Table 1.

Table 2 presents the composition of the experimental diets used in the study. Four diets were tested: (i) PC-100, a positive control meeting standard specifications for metabolizable energy (ME), calcium, phosphorus, and full mineral premix; (ii) NC-100, formulated with the same mineral premix as PC-100 but −100 kcal/kg ME, −0.05 percentage points calcium, and −0.10 percentage points phosphorus (dry matter [DM] basis), and supplemented with phytase (Ronozyme HiPhorius, 0.05 kg/ton, delivering ≈2,000 FTU/kg feed) and a enzymatic complex (Ronozyme VP, 0.30 kg/ton, delivering β-glucanase ≈1,500 U/kg and pectinase ≈15 U/kg); and (iii–iv) NC-67 and NC-34, which were derived from NC-100 by reducing the mineral premix inclusion by 33% and 66%, respectively, while keeping ME, calcium, phosphorus, and enzyme dosing identical to NC-100. Mineral premix was included at 0.07%, 0.07%, 0.05%, and 0.02% of the diet in PC-100, NC-100, NC-67, and NC-34, respectively (Supplement 1). Regarding the selenium, as specified by the manufacturer, the premix contained 0.250 mg selenium/kg (≈25 ppm). The calculated dietary selenium intake was 0.17, 0.17, 0.12, and 0.05 mg/kg for PC-100, NC-100, NC-67, and NC-34, respectively (dry basis). The daily selenium intake was estimated as dietary selenium_(mg/kg)_ × DMI_(kg/day)_ (Supplement 1). Pigs were assigned to the diets according to a randomized block design (3 blocks; 2 pigs per treatment per block). Feeding was adjusted based on body weight of 550 kcal ME/kg BW^0.6^/day [22].

### Procedures and sample collection

Pigs were weighed at the beginning and end of the balance trial, and average daily gain (ADG) was calculated by difference. The daily feed offered and refused was recorded to calculate daily feed intake (DFI), and feed efficiency (FE) was estimated by dividing ADG by DFI. During the 10-day period in the metabolic cages, pigs were fed twice daily (08:00 and 16:00 h). Water was provided ad libitum during the first 5 days (adaptation period). During the 5-days balance period (day 6–10), pigs received a standardized water allowance of 3 L per kg dry matter intake (DMI) to minimize wastage and ensure quantitative urine collection in metabolism crates. This allowance lies within typical water:feed ratios for grow-finish pigs (≈2:1–3:1) and is commonly used to reduce polydipsia and spillage during collection phases. Animals were checked twice daily for hydration status and behavior; no abnormalities were observed [23,24].

From day 6 to day 10, total feces and urine were collected to determine the balance of macrominerals (calcium, phosphorus, potassium, magnesium, sodium, and sulfur), microminerals (iron, zinc, manganese, and copper), and energy. On day 6, each pig received 3 g of ferric oxide (as an indigestible marker) mixed into 100 g of feed. The remaining feed was offered after the marked portion was consumed. Fecal collection began with the first appearance of the marker in the feces. On the morning of day 10, pigs again received 100 g of marked feed, as previously described, and fecal collection ended upon reappearance of the marker. Feces were stored at −20°C.

Urine was collected twice daily over the 5-day period. The collection container contained 40 mL of 6M HCl to acidify the urine and prevent ammonia volatilization. Urine collected over a 24-h period was filtered through cheesecloth and glass wool, weighed, and a 10% aliquot was stored at −20°C until analysis.

### Laboratory analyses

Fecal samples were dried in a forced-air oven at 55°C for 48 h, then homogenized and ground through a 0.5 mm mesh screen using a laboratory mill (Arthur H. Thomas). In both, experimental diets and fecal samples, DM, crude protein (AOAC methods 934.01 and 976.05; AOAC International [25]), and gross energy were analyzed using an adiabatic bomb calorimeter (model 1281; Parr). Minerals in feed and feces were determined by ICP-OES following microwave-assisted acid digestion (HNO_3_/H_2_O_2_) and calibration with traceable multi-element standards; reagent blanks, duplicates, and spike recoveries were run (RPD<10%; 90%–110%). Urine samples were thawed, homogenized, and subsampled for the same determinations.

For energy determination, 10 mL aliquots per experimental unit were dispensed into pre-weighed and tared 5×7.5-cm polyethylene bags (S-940; 2 mil), placed on plastic trays, and frozen at −45°C prior to lyophilization. After freeze-drying, samples were stored in desiccators until processing. Thirty minutes post-removal, the final weight of each bag plus sample was recorded to calculate DM percentage by difference from the initial weight. Gross energy content of the lyophilized urine was estimated according to Le Bellego et al [26].

### Calculations

Based on DFI, the intake of DM (g/day), macrominerals (g/day), microminerals (mg/day), and energy (kcal/day) was estimated by multiplying feed intake by nutrient concentration in the diet. Fecal excretion of DM, macro- and microminerals, and energy was calculated by multiplying the amount of dry feces produced by the nutrient concentration in feces. Urinary excretion of nitrogen (g/day) and energy (kcal/day) was estimated by multiplying the total volume of urine produced by the nutrient concentration in urine. AFDof DM, macro- and microminerals, and energy was calculated using the equation proposed by Stein et al [27]:


(1) $$AFD(\%)=\left[\frac{\left(FI\times NC)-(AF\times CEN\right)}{FI\times NC}\right]\times 100,$$

where AFD = apparent fecal digestibility; FI = amount of feed intake (g); NC = nutrient concentration in the feed (g, mg, or kcal); AF = amount of feces (g); and CEN = concentration of excreted nutrient (g, mg, or kcal).

Mineral retention relative to absorbed minerals was estimated by dividing the retained mineral content by the difference between mineral intake and fecal mineral excretion. Digestible energy (DE) was calculated by subtracting fecal energy from gross energy intake. ME was calculated by subtracting both fecal and urinary energy (UE) from gross energy intake, and it was expressed as both, as percentage and as kcal/kg of feed.

### Statistical analysis

Data was analyzed using a randomized block design, with four treatments and six replicates per treatment, distributed across three blocks. The statistical model included the fixed effect of treatment and the random effect of block. The GLM procedure of SAS ver. 9.4 (SAS Institute) was used to perform an analysis of variance (ANOVA). The statistical model used was as follows:


(2) $$Y_{ijk}=\mu +B_{i}+T_{j}+{ɛ}_{ijk}$$

where: Y*_ijk_* = response variable; μ = overall population mean; B*_i_* = random effect of the *^i^*^-^th block, with *i* = 1, 2, and 3; T*_j_* = fixed effect of the *^j^*^-^th treatment, with *j* = 100%, 66%, and 33% mineral inclusion; ɛ*_ijk_* = random error associated with each observation (assumed to be independently and normally distributed: ~*NID* = 0, $\sigma_{\text{e}}^{2}$.

Least squares means (LSMeans) were used to estimate treatment means, and significance was declared at α≤0.05. Orthogonal linear and quadratic contrasts were applied to evaluate the effects of progressive reduction in mineral premix (100%, 66%, 33%) on the physiological variables measured. Results are reported as means±standard error of the mean (SEM). Tables include p-values for treatment effect (T) and, where appropriate, for linear (L) and quadratic (Q) trends.

To assess whether water allowance could bias ME through UE, we performed two sensitivity checks: (1) linear regression of UE concentration (kcal/L) on urine volume (L/day); and (2) linear regression of UE (kcal/day) on urine volume, including urinary nitrogen (g/day) as a covariate, given the established linear dependence of UE on urinary nitrogen.

## RESULTS

### Growth performance and mineral balance

The results for productive performance and nitrogen balance are presented in Table 3. No significant differences (p>0.05) were observed in initial or final body weight among treatments. ADG and FE showed similar values across groups (p>0.05), indicating that trace mineral reduction and enzyme supplementation did not compromise pig growth performance.

Regarding DM intake, no significant differences were observed among treatments (p = 0.80). However, fecal DM excretion was lower (p = 0.03) in NC-34 (183.64 g/day) compared to NC-100 (212.65 g/day), reflecting a trend toward higher AFD of DM in NC-34 (88.28%) relative to NC-100 (86.54%) (p = 0.08). This responses exhibited a significant quadratic effect (p = 0.04; Table 3).

Nitrogen balance differed among treatments. Although nitrogen intake, absorption, and retention did not differ among treatments (p>0.05), urinary nitrogen excretion was lower in NC-34 (5.72 g/day) compared to the other treatments (p< 0.01; Table 3). This lead to reduced total nitrogen excretion (10.72 g/day; p<0.01) and increased retention efficiency. The proportion of retained nitrogen relative to absorbed nitrogen was higher in NC-34 (81.69%) compared to NC-100 (77.00%) and PC-100 (73.46%) (p<0.01), suggesting improved physiological efficiency of nitrogen utilization when trace mineral inclusion was reduced and exogenous enzymes were added.

Regarding macromineral balance, Table 4 presents the data for calcium, phosphorus, potassium, magnesium, sodium, and sulfur. Calcium intake decreased as mineral inclusion was reduced (PC-100: 19.91–NC-34: 17.90 g/day; p<0.01), and the same pattern was observed for fecal calcium (PC-100: 4.57–NC-34 3.79 g/day; p<0.01) and for calcium retention (PC-100: 15.33–NC-34 14.11 g/day; p<0.01). In contrast, AFD of calcium did not differ among treatments (mean ≈ 78%; p = 0.28). For phosphorus, intake, fecal excretion, and retention all differed among treatments (p<0.01). AFD of phosphorus was higher with the enzyme-supplemented moderate reductions (NC-100 74.89%, NC-67 75.21%) than in PC-100 (69.15%; p<0.01), whereas NC-34 (74.98%) was statistically like PC-100. Consistent with this, phosphorus retention was greatest in NC-100 (5.74 g/day) and NC-67 (5.68 g/day), with NC-34 (5.56 g/day) having an intermediate level that did not differ from PC-100 (5.40 g/day).

For potassium, intake, excretion, and AFD differed significantly among treatments (p<0.001; Table 4). AFD increased progressively as mineral inclusion was reduced, reaching the highest value in NC-34 (81.43%) compared to PC-100 and NC-100 (76.34% and 76.28%, respectively). In case of magnesium, although intake and fecal excretion decreased with mineral reduction (p<0.001; Table 4), AFD did not differ significantly among treatments (p = 0.53), showing mean values close to 51%. Sodium exhibited a clear response: despite lower intake in NC-34 (2.30 g/day; p<0.05), AFD was higher (83.56%) than in PC-100 (80.67%; p = 0.05). Total sodium retention was also lower in NC-34 (1.92 g/day; Table 4). Regarding sulfur balance, both intake and fecal excretion decreased with mineral reduction (p<0.01; Table 4). AFD of sulfur increased progressively from 55.85% in PC-100 to 60.73% in NC-34, although the differences were not statistically significant (p = 0.81; Table 4).

Regarding trace mineral balance, Table 5 shows the effect of progressive mineral reduction and exogenous enzyme supplementation on the balance and AFD of iron, zinc, manganese, and copper. Iron intake was significantly reduced (p< 0.01), from 776.17 mg/day in PC-100 to 490.88 mg/day in NC-34. Fecal excretion of iron also decreased (from 428.65 to 252.10 mg/day; p<0.01), resulting in lower total retention (from 347.52 to 238.78 mg/day; Table 5). AFD of iron did not differ significantly among treatments (p = 0.50; Table 5).

For zinc, intake, fecal excretion, and retention varied significantly among treatments (p<0.01; Table 5). AFD of zinc improved as mineral inclusion levels decreased, reaching the highest value in NC-34 (50.01%) compared to PC-100 (40.62%; p<0.01), suggesting greater absorption efficiency at lower supplementation levels. For manganese, a progressive decrease in intake and fecal excretion was observed as mineral inclusion decreased (p<0.01; Table 5). However, AFD of manganese did not differ among treatments (p = 0.96), suggesting that reduced inclusion did not impair its intestinal availability. Copper intake and fecal excretion differed among treatments (p<0.01; Table 5), and AFD increased from 35.46% (PC-100) to 48.39% (NC-34; p<0.01) as mineral inclusion decreased. Copper retention was higher in NC-100 and NC-67, but decreased in NC-34 (p = 0.02), indicating that retention peaked at intermediate inclusion levels while excess excretion increased at higher or lower supplementation.

### Energy balance

For energy balance, Table 6 shows the effect of mineral reduction and exogenous enzyme supplementation on energy-related parameters. No significant differences (p>0.05) were observed in energy intake among treatments, with average values ranging from 6,289 to 6,354 kcal/day. Fecal energy showed a decreasing trend as mineral inclusion was reduced, with the lowest value observed in NC-34 (313.32 kcal/day) and the highest in NC-100 (356.61 kcal/day). Although this difference did not reach statistical significance (p = 0.08), the linear trend was significant (p = 0.02), suggesting a favorable effect of mineral reduction on energy digestibility. Despite variations in fecal excretion, DE remained stable across treatments (ranging from 88.10% to 89.17%; p = 0.81). No significant differences were observed in UE or total excreted energy (p>0.05; Table 6).

ME values were slightly higher in NC-67 and NC-34 (87.50% and 87.66%, respectively), but not significantly different from the control groups (p = 0.11; Table 6). In terms of kcal/kg, NC-34 showed the highest energy availability (3,630 kcal/kg), numerically greater than NC-100 (3,593 kcal/kg), although the difference was not statistically significant (p = 0.06). Overall, these results suggest that the strategic reduction of trace minerals, when combined with exogenous enzymes, does not compromise the energy utilization of the diet and may even enhance fecal digestive efficiency.

### Sensitivity analysis

**Figure 1:**
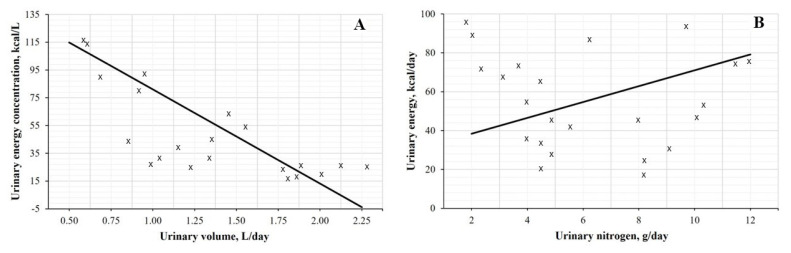
Sensitivity of urinary energy to urine volume and urinary nitrogen during the 5-day balance period. (A) Regression of urinary energy concentration (kcal/L) on urine volume (L/d): *Y* = 148.80–67.82*X*; *r* = −0.77; p<0.01, where *Y*: Urinary energy concentration and *X*: Urinary volume. (B) Regression of daily urinary energy (kcal/d) on urinary nitrogen (g/d) and urine volume (L/d): *Y* = 83.45+4.09*X*_1_–39.48*X*_2_; *r* = 0.41; p = 0.39, where *Y*: Urinary energy, *X*_1_: Urinary nitrogen and X_2_: Urinary volume.

UE concentration was negatively associated with urine volume (*Y* = 148.80 – 67.82*X*; *r* = −0.77; p<0.01) (Figure 1A), indicating that higher urine output diluted the energy content. In contrast, daily UE was weakly related to urinary nitrogen and urine volume (*Y* = 83.45+4.09*X*_1_–39.48*X*_2_; *r* = 0.41; p = 0.39) (Figure 1B), showing no significant association with nitrogen excretion.

## DISCUSSION

The results show that a strategic nutrient reduction—using an enzyme-supplemented diet with graded reductions of the trace-mineral premix—combined with phytase, pectinase, and β-glucanase improves nutrient-utilization efficiency in growing pigs, particularly for nitrogen and trace minerals, without compromising ADG or FE during the 10-day mineral–energy balance.

This performance neutrality is consistent with Harper et al [28] and Valente et al [12], where enzyme supplementation enhanced digestibility and bioavailability and enabled nutrient-sparing formulations. In contrast, within the same window the reduced-mineral diets lowered calcium retention, indicating reduced net mineral accretion during collection. Taken together, the dataset supports an efficiency-driven, enzyme-assisted approach in line with precision nutrition [1], while explicitly limiting inference to the short period tested.

Crucially, across NC-100, NC-67, and NC-34 the dietary concentrations of calcium and phosphorus were held constant; hence treatment effects on daily calcium and phosphorus flows reflect feed intake and enzymatic availability, not changes in formulated supply. In agreement with Table 4, calcium intake declined relative to PC-100 and calcium retention decreased in parallel, whereas AFD of calcium remained unchanged (≈ 78%; p = 0.28). The stable calcium digestibility confirms preserved absorption, so the reduction in calcium retention is a quantity effect driven by intake. For phosphorus, all flows differed among treatments; phosphorus digestibility was higher with the enzyme-supplemented moderate reductions (NC-100/NC-67) than in PC-100, with NC-34 statistically similar to PC-100. Consistently, phosphorus retention ranked NC-100 ≈ NC-67>NC-34≥PC-100, showing that phytase-based supplementation improved phosphorus uses at constant dietary phosphorus.

Within this balance window, DM digestibility and relative nitrogen retention increased as microminerals were progressively reduced, with the greatest response in NC-34. This pattern fits a synergistic, enzyme-assisted nutrient-sparing effect, whereby phytase, pectinase, and β-glucanase hydrolyze phytate and non-starch polysaccharides, improving the release and absorption of amino acids and minerals [29,30]. Concomitantly, urinary and total nitrogen excretion decreased while net nitrogen retention was maintained, indicating improved metabolic efficiency of absorbed nitrogen [31]. At the intestinal level, increased digestibility aligns with improvements in villus morphology and functionality reported with enzyme supplementation [12,32], greater absorptive capacity and epithelial integrity, and reduced protein fermentation in the large intestine—hence lower production of amines, indoles, and ammonia [33].

For macrominerals beyond phosphorus, sodium and potassium digestibility were also higher in enzyme-supplemented treatments (notably NC-34), which is consistent with reduced ionic competition and improved epithelial function under enzyme action [34,35]. For trace minerals, zinc and copper digestibility increased as dietary inclusion decreased, most clearly in NC-34, a pattern aligned with autoregulatory down-modulation of intestinal uptake at high dietary zinc and copper [36,37] and with lower transporter saturation (e.g., DMT1, divalent metal transporter–1) and diminished cationic antagonism [38].

Although digestible and ME did not differ significantly among treatments, a positive pattern was evident in the reduced-energy, enzyme-supplemented diets—again strongest in NC-34—indicating that enzymes-maintained energy-utilization efficiency under restriction [39]. Mechanistically, this agrees with enhanced fermentation of soluble fiber and lower energy losses via gas or fecal excretion of undigested compounds [40].

Within this controlled, short-term study, ME did not differ among treatments, and indices of DM digestibility and relative nitrogen retention showed favorable patterns under enzyme-assisted premix reduction. Taken together, these findings suggest potential for more efficient and environmentally responsible formulations in swine production—through reduced reliance on inorganic minerals and the use of exogenous enzymes to improve nutrient bioavailability—without compromising short-term productive performance. However, results should be interpreted conservatively: they document responses under controlled conditions and require confirmation in longer-duration studies conducted in commercial environments, including hot-season scenarios with variable water intake, before broader inferences are made.

### Limitations and methodological considerations

This was a short-term (10-day) balance study conducted in metabolism crates under controlled conditions; therefore, outcomes may not extrapolate to longer production phases or heavier body weights. During the 5-day balance period a fixed water allowance (3 L/kg DMI) was used to ensure quantitative urine collection; while standard for metabolism work, this differs from commercial settings, particularly in hot seasons when pigs commonly reduce feed intake and increase water intake. Findings should thus be interpreted conservatively: they document short-term nutrient-use responses under controlled conditions and do not imply identical magnitudes or durability under field environments.

Regarding selenium, PC-100 and NC-100 were within NRC [21] recommendations, whereas NC-67 and NC-34 were lower; no clinical signs were observed over 10 days, but the absence of antioxidant biomarkers (e.g., plasma glutathione peroxidase) is a limitation that should be addressed in future work. Overall, these data provide proof-of-concept and should be validated in longer, commercial-environment trials (including hot-season scenarios with variable water intake) before broader inferences are made.

## CONCLUSION

In this 10-day balance trial with growing pigs, enzyme-assisted reductions of the trace-mineral premix maintained ADG and FE and did not change ME across treatments. Within this window, we observed higher DM digestibility (trend) and greater relative nitrogen retention, together with lower urinary/total nitrogen excretion, while calcium retention decreased as mineral inclusion was reduced. These results support the short-term feasibility of a nutrient-sparing, enzyme-assisted strategy without performance loss, while confirming that effects on mineral accretion warrant validation in longer, commercial-environment studies before broader inferences are made.

## Figures and Tables

**Table 1:** Description of the experimental treatments

	PC-100	NC-100	NC-67	NC-34
ME (Mcal/kg)	3.30	3.20	3.20	3.20
Digestible phosphorus (% of diet, DM)^1)^	0.45	0.35	0.35	0.35
Coverage vs. NRC digestible phosphorus (%)^2)^	100	77.8	77.8	77.8
Phytase inclusion (kg/t)^3)^	-	0.05	0.05	0.05
Phytase activity (FTU/kg feed)^3)^	-	2,000	2,000	2,000
Enzyme complex (kg/ton)^4)^	-	0.30	0.30	0.30
β-glucanase (U/kg feed)^4)^		1,500	1,500	1,500
Pectinase (U/kg feed)^4)^		15	15	15
Mineral premix inclusion (%)	100	100	66	33

Dietary enzyme activities are expressed per kg of complete feed.

1) Digestible phosphorus (% DM): absolute values shown assuming NRC [21] digestible phosphorus requirement = 0.45% for ~50 kg pigs; NC diets set at 0.35% (NRC–0.10 percentage points).

2) Coverage vs. NRC digestible phosphorus (%): values computed using NRC [21] digestible phosphorus requirement = 0.45% for ~50 kg pigs; coverage = 100×(Dietary digestible phosphorus / NRC digestible phosphorus).

3) Phytase: Ronozyme HiPhorius (DSM Nutritional Products México), a 6-phytase encoded by a *Citrobacter braakii* gene and expressed in *Aspergillus oryzae*. At an inclusion of 0.05 kg/ton (0.05 g/kg), and a potency of 40,000 FYT/g (HiPhorius 40), dietary activity equals approximately 2,000 FTU/kg feed (FTU/FYT defined as the amount of enzyme that releases 1 μmol inorganic phosphate per minute from sodium phytate at defined pH/temperature).

4) Enzyme complex: Ronozyme VP (DSM Nutritional Products México; *Aspergillus aculeatus*). Label activities per gram of product: β-glucanase 5,000 U/g; pectinase 50 U/g. With an inclusion of 0.30 kg/ton (0.30 g/kg), the calculated dietary activities are: β-glucanase 1,500 U/kg feed and pectinase 15 U/kg feed.

PC, positive control; NC, negative control; ME, metabolizable energy; DM, dry matter.

**Table 2:** Ingredient composition and nutritional contribution of the experimental diets

Ingredients	PC-100	NC-100	NC-67	NC-34
Sorghum grain (8.5%)^1)^	73.65	73.59	73.60	73.63
Soybean meal (46%)^1)^	18.83	14.04	14.04	14.04
Canola meal (36%)^1)^	-	8.00	8.00	8.00
Tallow	4.00	1.50	1.50	1.50
Mono- and dicalcium phosphate	1.29	0.73	0.73	0.73
Calcium carbonate	0.80	0.80	0.80	0.80
L-Lysine HCl	0.43	0.43	0.43	0.43
Iodized salt	0.36	0.36	0.36	0.36
L-Threonine	0.13	0.11	0.11	0.11
Choline HCl	0.13	0.13	0.13	0.13
Trace mineral premix^2)^	0.07	0.07	0.05	0.02
Chelating agent	0.15	0.15	0.15	0.15
DL-Methionine	0.12	0.04	0.04	0.04
Vitamin premix^3)^	0.05	0.05	0.05	0.05
Total	100.00	100.00	100.00	100.00
Nutritional contribution				
ME (Mcal/kg)	3.30	3.20	3.20	3.20
Crude protein (%)	15.35	15.93	15.93	15.93
Total lysine (%)	1.03	1.05	1.05	1.05
Digestible lysine (%)	0.93	0.93	0.93	0.93
Total Ca (%)	0.60	0.55	0.55	0.55
Total P (%)	0.51	0.46	0.46	0.46
Available P (%)	0.31	0.21	0.21	0.21

1) Values in parentheses indicate the crude protein content (%) of the ingredient as determined by proximate analysis.

2) The trace mineral premix provided the following per kg of feed: Co, 0.60 mg; Cu, 12 mg; Fe, 100 mg; I, 0.80 mg; Mn, 30 mg; Se, 0.25 mg; Zn, 120 mg.

3) The vitamin premix provided the following per kg of feed: Vitamin A, 13,300 IU; Vitamin D_3_, 3,700 IU; Vitamin E, 160 mg; Menadione, 9.40 mg; Biotin, 0.67 mg; Cyanocobalamin, 0.07 mg; Folic acid, 5.33 mg; Niacin, 66.70 mg; Pantothenic acid, 46.70 mg; Pyridoxine, 6.67 mg; Riboflavin, 12.00 mg; Thiamine, 4.00 mg; Vitamin C, 266.70 mg.

Source: DSM Mexico.

PC, positive control; NC, negative control; ME, metabolizable energy.

**Table 3:** Effect of mineral reduction and exogenous enzyme supplementation on growth performance, dry matter digestibility, and nitrogen balance in growing pigs

	Tratment (T)				SEM	p-value		
	
	PC-100	NC-100	NC-67	NC-34		T	L	Q
Initial body weight (BW) (kg)	48.85	49.83	49.28	49.07	0.63	0.72	0.97	0.64
Final body weight (BW) (kg)	51.12	52.10	51.61	51.30	0.65	0.71	0.96	0.59
Average daily gain (ADG) (g)^1)^	452.77	455.56	466.56	447.22	6.01	0.14	0.84	0.18
Feed efficiency (gain/feed) (kg/kg)	0.29	0.29	0.30	0.28	0.01	0.29	0.83	0.50
Dry matter intake (DMI) (g/day)	1,568.24	1,584.57	1,575.63	1,570.86	12.42	0.80	0.98	0.70
Dry matter excretion (g/day)	190.40^a^	212.65^b^	201.24^ab^	183.64^a^	16.51	0.03	0.34	0.02
Apparent fecal digestibility of DM (%)	87.81	86.54	87.06	88.28	0.51	0.08	0.412	0.04
Nitrogen (N) balance (g/day)								
N intake	37.11	37.69	36.66	35.99	0.64	0.29	0.13	0.19
Fecal N	5.61	5.61	5.41	5.00	0.23	0.19	0.04	0.09
Urinary N	8.05^a^	7.36^a^	7.02^a^	5.72^b^	0.46	0.01	<0.01	<0.01
Absorbed N	31.49	32.08	31.25	30.98	0.81	0.80	0.51	0.70
Total N excretion	13.66^a^	12.97^ab^	12.43^b^	10.72^c^	0.49	<0.01	<0.01	<0.01
Retained N	23.44	24.72	24.23	25.27	0.83	0.47	0.18	0.40
N retention (%)	62.22^a^	65.26^a^	65.84^ab^	70.11^b^	1.53	<0.01	<0.01	<0.01
N retention relative to absorbed N (%)	73.46^a^	77.00^a^	77.39^a^	81.69^b^	1.51	<0.01	<0.01	<0.01

PC-100 = positive control diet formulated according to NRC [21]; NC-100, NC-67, NC-34 = diets with reduced energy and mineral inclusion (100%, 66%, and 33% of the trace mineral premix, respectively) supplemented with enzymes (phytase, pectinase, and β-glucanase).

1) Weight gain considered only during the 5-days of the balance test.

a–c Different superscripts within a row indicate statistical differences (p<0.05).

SEM, standard error of the mean; L, linear effect; Q, quadratic effect.

**Table 4:** Effect of mineral reduction and exogenous enzyme supplementation on the balance and apparent fecal digestibility of macrominerals in growing pigs

	Treatment (T)				SEM	p - value		
	
	PC-100	NC-100	NC-67	NC-34		T	L	Q
Calcium (Ca) balance (g/d)								
Ca intake	19.91^a^	18.25^b^	17.73^b^	17.90^b^	0.17	<0.01	<0.01	0.01
Fecal Ca	4.57^a^	3.86^b^	3.80^b^	3.79^b^	0.11	<0.01	<0.01	<0.01
Ca retention	15.33^a^	14.39^b^	13.94^b^	14.11^b^	0.23	<0.001	<0.01	<0.01
Apparent fecal digestibility (%)	76.87	78.83	78.62	78.81	0.72	0.28	0.47	0.44
Phosphorus (P) balance (g/d)								
P intake	7.80^a^	7.64^ab^	7.55^bc^	7.34^c^	0.10	<0.01	<0.01	<0.01
Fecal P	2.40^a^	2.11^b^	1.86^c^	1.79^c^	0.07	<0.01	<0.01	<0.01
P retention	5.40^a^	5.74^b^	5.68^bc^	5.56^ac^	0.13	<0.01	<0.01	<0.01
Apparent fecal digestibility (%)	69.15^a^	74.89^b^	75.21^b^	74.98^a^	1.04	<0.01	<0.01	0.32
Potassium (K) balance (g/d)								
K intake	13.74^a^	13.36^b^	13.02^c^	12.47^d^	0.11	<0.01	<0.01	<0.01
Fecal K	3.26^a^	3.16^a^	2.74^b^	2.30^c^	0.11	<0.01	<0.01	<0.01
K retention	10.48	10.20	10.28	10.17	0.16	0.51	0.24	0.43
Apparent fecal digestibility (%)	76.34^a^	76.28^a^	78.80^b^	81.43^c^	0.89	<0.01	<0.01	<0.01
Magnesium (Mg) balance (g/d)								
Mg intake	5.48^a^	4.96^b^	4.88^b^	4.71^c^	0.52	<0.01	<0.01	<0.01
Fecal Mg	2.71^a^	2.41^b^	2.36^b^	2.18^b^	0.07	<0.01	<0.01	<0.01
Mg retention	2.78	2.56	2.52	2.53	0.10	0.27	0.10	0.15
Apparent fecal digestibility (%)	49.85	51.36	51.08	53.41	1.72	0.53	0.18	0.39
Sodium (Na) balance (g/d)								
Na intake	2.82^a^	2.68^b^	2.57^c^	2.30^d^	0.04	<0.01	<0.01	<0.01
Fecal Na	0.53^a^	0.44^b^	0.37^c^	0.38^c^	0.03	<0.01	<0.01	<0.01
Na retention	2.28^a^	2.24^a^	2.19^a^	1.92^b^	0.05	<0.01	<0.01	<0.01
Apparent fecal digestibility (%)	80.67^a^	83.21^ab^	85.24^b^	83.56^b^	1.15	0.04	0.04	0.02
Sulfur (S) balance (g/d)								
S intake	1.41^a^	1.11^b^	0.84^c^	0.78^c^	0.05	<0.01	<0.01	<0.01
Fecal S	0.60^a^	0.43^b^	0.31^c^	0.22^d^	0.02	<0.01	<0.01	<0.01
S retention	0.81^a^	0.68^ab^	0.53^b^	0.55^b^	0.06	<0.01	<0.01	<0.01
Apparent fecal digestibility (%)	55.85	58.50	59.39	60.73	2.85	0.81	0.32	0.62

PC-100 = positive control diet formulated according to NRC [21]; NC-100, NC-67, NC-34 = diets with reduced energy and mineral inclusion (100%, 66%, and 33% of the mineral premix, respectively) supplemented with enzymes (phytase, pectinase, and β-glucanase).

a–d Different superscript letters within a row indicate significant differences (p<0.05).

SEM, standard error of the mean; L, linear effect; Q, quadratic effect.

**Table 5:** Effect of mineral reduction and the use of exogenous enzymes on the balance and apparent fecal digestibility of trace minerals in growing pigs

	Treatment (T)				SEM	p-value		
	
	PC-100	NC-100	NC-67	NC-34		T	L	Q
Iron (Fe) balance (mg/d)								
Fe intake	776.17^a^	661.71^b^	514.47^c^	490.88^c^	15.48	<0.01	<0.01	<0.01
Fe in feces	428.65^a^	351.93^b^	274.93^c^	252.10^c^	12.27	<0.01	<0.01	<0.01
Fe retained	347.52^a^	309.78^b^	239.53^c^	238.78^c^	13.11	<0.01	<0.01	<0.01
Apparent fecal digestibility (%)	44.78	45.51	45.79	48.55	1.79	0.50	0.19	0.40
Zinc (Zn) balance (mg/d)								
Zn intake	215.50^a^	209.05^b^	160.39^c^	130.20^d^	2.60	<0.01	<0.01	<0.01
Zn in feces	128.50^a^	122.37^b^	90.51^c^	55.17^d^	3.96	<0.01	<0.01	<0.01
Zn retained	86.99^a^	86.68^a^	69.87^b^	75.02^b^	4.20	0.01	0.01	0.02
Apparent fecal digestibility (%)	40.62^a^	41.35^a^	43.18^a^	50.01^b^	2.12	<0.01	<0.01	<0.01
Manganese (Mn) balance (mg/d)								
Mn intake	57.98^a^	51.02^b^	38.74^c^	31.00^d^	1.22	<0.01	<0.01	<0.01
Mn in feces	31.39^a^	27.92^a^	21.03^b^	16.50^c^	1.33	<0.01	<0.01	<0.01
Mn retained	26.59^a^	23.10^b^	17.71^c^	14.49^c^	1.15	<0.01	<0.01	<0.01
Apparent fecal digestibility (%)	45.72	46.15	45.67	47.22	2.34	0.96	0.69	0.90
Copper (Cu) balance (mg/d)								
Cu intake	21.30^a^	20.16^a^	16.97^b^	11.41^c^	1.02	<0.01	<0.01	<0.01
Cu in feces	12.92^a^	11.16^b^	9.06^c^	5.81^d^	0.48	<0.01	<0.01	<0.01
Cu retained	8.38^a^	8.99^a^	7.91^a^	5.60^b^	0.80	0.02	0.01	0.01
Apparent fecal digestibility (%)	35.46^a^	41.30^ab^	45.62^b^	48.39^b^	2.62	<0.01	<0.01	0.01

PC-100 = positive control diet formulated according to NRC [21]; NC-100, NC-67, NC-34: diets with reduced energy and mineral inclusion (100%, 66%, and 33% of the trace mineral premix, respectively), supplemented with enzymes (phytase, pectinase, and β-glucanase).

a–d Different superscript letters within a row indicate statistical difference (p<0.05).

SEM, standard error of the mean; L, linear effect; C, quadratic effect.

**Table 6:** Effect of mineral reduction and exogenous enzyme supplementation on energy balance in growing pigs

	Treatment (T)				SEM	p-value		
	
	PC-100	NC-100	NC-67	NC-34		T	L	Q
Energy intake (kcal/d)	6,333	6,354	6,314	6,289	48.63	0.81	0.43	0.65
Fecal energy (kcal/d)	347.02	356.61	324.25	313.32	13.09	0.08	0.02	0.06
Digestible energy (%)	89.11	88.10	88.91	89.17	0.34	0.81	0.41	0.68
Digestible energy (kcal/kg)	3,692	3,653	3,684	3,690	11.22	0.51	0.62	0.12
Urinary energy (kcal/d)	50.14	60.73	56.54	60.66	4.30	0.26	0.16	0.28
Excreted energy (kcal/d)	397.16	417.34	380.79	373.99	13.51	0.11	0.08	0.13
Metabolizable energy (%)	87.19	86.59	87.50	87.66	0.33	0.11	0.12	0.16
Metabolizable energy (kcal/kg)	3,642	3,593	3,627	3,630	11.64	0.06	0.97	0.09

PC-100 = positive control diet formulated according to NRC [21]; NC-100, NC-67, NC-34: diets with reduced inclusion of energy and minerals (100%, 66%, and 33% of the mineral premix, respectively) supplemented with enzymes (phytase, pectinase, and β-glucanase).

SEM, standard error of the mean; L, linear effect; Q, quadratic effect.

## SUPPLEMENTARY MATERIAL

**Supplement 1**. Nutrient composition of experimental diets (analyzed; dry-matter basis).
