# Supplementary material for: Effect of strategic nutrient reduction and exogenous enzyme supplementation on mineral and energy balance in growing pigs
Source: Anim Biosci. 2025 Sep 30;39(2):250568. doi: 10.5713/ab.25.0568 (PMC12877397; doi:10.5713/ab.25.0568)
Supplement: Supplementary file 1 [file ab-25-0568-Supplementary-1.pdf]

Supplement 1. Nutrient composition of experimental diets (analyzed; dry-matter basis).

Nutrient	PC-100	NC-100	NC-67	NC-34
ME, kcal/kg.	3.30	3.20	3.20	3.20
Crude protein, %	15.35	15.93	15.93	15.93
Total Nitrogen, %	2.37	2.38	2.33	2.29
Total Phosphorus, %	0.50	0.48	0.46	0.40
Sulfur, %	0.09	0.07	0.05	0.05
Potassium, %	0.88	0.84	0.83	0.79
Calcium, %	1.27	1.15	1.09	1.01
Magnesium, %	0.35	0.31	0.31	0.30
Sodium, %	0.18	0.17	0.16	0.15
Iron, ppm	495.63	418.99	326.29	312.55
Zinc, ppm	137.44	131.88	101.88	82.65
Manganese, ppm	36.83	32.04	24.50	19.73
Copper, ppm	13.44	12.56	10.67	7.27
Selenium, mg/kg diet [&]	0.17	0.17	0.12	0.05
Selenium intake, mg/kg*	0.27	0.27	0.19	0.08

Values are on a DM basis. CP by Kjeldahl; minerals by ICP-OES. Analyzed ME (kcal/kg DM) derived from feed bomb calorimetry.

[&]According to the manufacturer's specification, the premix contained 0.25 mg Se/kg premix. The resulting dietary selenium contents were 0.175, 0.175, 0.125, and 0.05 mg/kg DM for PC-100, NC-100, NC-67, and NC-34, respectively, calculated as: $Se_{(mg/kg\ diet)} = \frac{premix\ inclusion,\%}{100} \times Se\ in\ premix_{(mg/kg)}$.

*Daily Se intake was estimated as $dietary\ Se_{(mg/kg)} \times DMI_{(kg/day)}$.
